# A Cell-Based Screen Reveals that the Albendazole Metabolite, Albendazole Sulfone, Targets *Wolbachia*

**DOI:** 10.1371/journal.ppat.1002922

**Published:** 2012-09-20

**Authors:** Laura R. Serbus, Frederic Landmann, Walter M. Bray, Pamela M. White, Jordan Ruybal, R. Scott Lokey, Alain Debec, William Sullivan

**Affiliations:** 1 Molecular, Cell and Developmental Biology, University of California, Santa Cruz, California, United States of America; 2 Department of Chemistry and Biochemistry, University of California, Santa Cruz, California, United States of America; 3 Polarity and Morphogenesis Group, Jacques Monod Institute, CNRS, University Paris Diderot, UPMC Bâtiment Buffon, Paris, France; Stanford University, United States of America

## Abstract

*Wolbachia* endosymbionts carried by filarial nematodes give rise to the neglected diseases African river blindness and lymphatic filariasis afflicting millions worldwide. Here we identify new *Wolbachia*-disrupting compounds by conducting high-throughput cell-based chemical screens using a *Wolbachia*-infected, fluorescently labeled *Drosophila* cell line. This screen yielded several *Wolbachia*-disrupting compounds including three that resembled Albendazole, a widely used anthelmintic drug that targets nematode microtubules. Follow-up studies demonstrate that a common Albendazole metabolite, Albendazole sulfone, reduces intracellular *Wolbachia* titer both in *Drosophila melanogaster* and *Brugia malayi*, the nematode responsible for lymphatic filariasis. Significantly, Albendazole sulfone does not disrupt *Drosophila* microtubule organization, suggesting that this compound reduces titer through direct targeting of *Wolbachia*. Accordingly, both DNA staining and FtsZ immunofluorescence demonstrates that Albendazole sulfone treatment induces *Wolbachia* elongation, a phenotype indicative of binary fission defects. This suggests that the efficacy of Albendazole in treating filarial nematode-based diseases is attributable to dual targeting of nematode microtubules and their *Wolbachia* endosymbionts.

## Author Summary

*Wolbachia*-based neglected diseases currently threaten one sixth of the world population. Millions of people are infected with filarial nematodes that rely upon endosymbiotic *Wolbachia* bacteria for their survival. These *Wolbachia* ultimately induce an immune response that gives rise to African river blindness or lymphatic filariasis. Thus, targeting *Wolbachia* will prevent induction of disease symptoms while also eliminating the filarial nematode infection. To identify new, fast-acting anti-*Wolbachia* drugs, we tested candidate compounds in *Wolbachia*-infected insect cells using automated robotics. Some of the anti-*Wolbachia* compounds we discovered closely resembled Albendazole, an FDA-approved drug already used worldwide to combat filarial nematode infections. We found that a common Albendazole metabolite strongly suppresses *Wolbachia* density in fruit flies and filarial nematodes by disrupting *Wolbachia* growth. These findings suggest that Albendazole is effective in treating filarial nematode-based diseases because it independently targets both the nematode and its essential *Wolbachia* endosymbionts. This has immediate implications for treating lymphatic filariasis and African river blindness.

## Introduction

*Wolbachia* are intracellular maternally transmitted bacteria present in the majority of all insect species as well as some mites, crustaceans and filarial nematodes [1], [2]. *Wolbachia* were initially studied in insects because they induce unconventional reproductive phenotypes including sperm-egg cytoplasmic incompatibility, feminization of males, male-killing, and parthenogenesis [3], [4]. *Wolbachia* are essential endosymbionts of some filarial nematodes and recent studies demonstrated that they are the causative agent of African river blindness and also contribute to lymphatic filariasis [5], [6]. One sixth of the world population is at risk of infection by *Wuchereria bancrofti*, *Brugia timori* and *Brugia malayi*, the filarial nematode species that cause lymphatic filariasis [7]. *Wolbachia* released from filarial nematodes into the human body trigger an inflammatory reaction that underlies the lymphedema and corneal occlusion associated with these neglected diseases [8], [9], [10], [11], [12], [13], [14], [15], [16], [17], [18].

Lymphatic filariasis and African river blindness have traditionally been treated through the administration of three drugs, singly or in combination: diethylcarbamazine (DEC), ivermectin (IVM) and albendazole (ALB). These drugs target the filarial nematodes associated with these diseases, namely *Onchocerca volvulus*, *B. timori*, *B. malayi*, and *W. bancrofti* [5], [6], [19]. DEC disrupts the nematodes by targeting the arachidonic acid metabolic pathway in the host [20]. IVM disrupts glutamate-gated chloride channels in the nematode that control release of excretory/secretory vesicles that would normally suppress the immune response [21], [22]. ALB is a benzimidazole used to disrupt the nematode microtubule cytoskeleton [23]. Orally administered ALB is rapidly metabolized by in the intestinal mucosa and liver into albendazole sulfoxide (ALB-SO) and albendazole sulfone (ALB-SO2) [24], [25]. ALB-SO is normally considered to be the “active,” form of Albendazole against systemic parasites, while ALB-SO2 is considered to be an inactive form of the drug [26]. All three drugs exhibit microfilaricidal effects [19]. The macrofilaricidal effects of ALB are not clear, though specific formulations induce worm sterility in animal models [27]. In addition, a number of clinical trials demonstrate that ALB when used in combination with DEC or IVM is macrofilaricidal [28], [29], [30], [31], [32].

*Wolbachia* are obligate symbionts of filarial nematodes required for normal embryogenesis, larval development and perhaps most significantly adult survival [33], [34], [35], [36], [37], [38], [39], [40], [41]. A recent study demonstrated that loss of *Wolbachia* in the adult results in high levels of apoptosis throughout the nematode [37]. Studies have also found that much of the pathology associated with filarial nematode diseases is due to induction of innate and adaptive host immune responses upon release of *Wolbachia* from their nematode hosts [8], [9], [10], [11], [12], [13], [14], [15], [16], [17], [18]. These discoveries suggest that compounds directly targeting *Wolbachia* may be a powerful alternative to the more traditional approaches for treating these diseases. The major advantage of this approach is that it targets adults as well as microfilaria and the *Wolbachia* will be eliminated prior to death of the nematode, reducing the destructive effects of the human immune response. In addition, loss of *Wolbachia* leads to a slow death of the adults, providing time for the infected individual to clear the dead nematodes without deleterious side effects [35], [42], [43], [44], [45], [46], [47]. Finally, antihelminthic drugs such as ivermectin, administered to patients co-infected with *Loa Loa* nematodes, can potentially trigger lethal encephalitis [48]. *Loa loa* does not require or maintain *Wolbachia* and thus will not be affected by anti-*Wolbachia* therapies, thereby avoiding these deleterious side effects [49]. The promise of the anti-*Wolbachia* based therapies in combating lymphatic filariasis has been demonstrated in clinical trials in which daily doses of doxycycline (DOX) for 4 weeks resulted in nematode sterility and death [6]. In addition, the pathologies associated with the infection, lymphedema and hydrocele, were dramatically reduced [8], [9].

These studies also revealed that a three-week course of DOX was insufficient to produce significant mortality of the adult nematodes, highlighting the need to identify more potent anti-*Wolbachia* compounds [50]. To this end, we generated a *Wolbachia-*infected *Drosophila* cell line and conducted an automated, cell-based screen to identify lead compounds that reduced intracellular *Wolbachia* infection. This screen of two libraries totaling 4926 compounds yielded 40 anti-*Wolbachia* compounds, including several that structurally resembled ALB. Our follow-up testing indicated that ALB-SO2 directly targets *Brugia* by depolymerizing its microtubules. Here we demonstrate that ALB-SO2 also disrupts *Brugia Wolbachia* independently of its effects on the *Brugia* cytoskeleton. Furthermore, ALB-SO2 treatment of *Brugia* led to visibly elongated *Wolbachia* morphology indicative of a binary fission failure, consistent with a direct impact of ALB-SO2 upon *B. malayi Wolbachia*.

## Results

### Development of a *Drosophila* cell line constitutively infected with *Wolbachia*

To identify compounds that affect intracellular *Wolbachia* titer, we generated new *Drosophila* tissue culture cells constitutively infected with *Wolbachia* [51] (see Methods). There is currently no stable nematode tissue culture line, nor any type of cell line constitutively infected with *Wolbachia* derived from filarial nematodes. *Wolbachia*-infected insect cells provide an opportunity to identify drugs that disrupt *Wolbachia* through conserved molecular pathways. The cell line used for this study, JW18, is particularly amenable to high throughput screening as *Wolbachia* are maintained in approximately 90% or more of the host cell population (n = 1053 cells scored). The *Wolbachia* load in 6.7% of infected cells ranges from 1–46 bacteria, while *Wolbachia* load in the other 93% of infected cells is obscured by crowding of the bacteria (n = 205 cells scored). The mitotic index of JW18 and tetracycline-cured JW18 cells, henceforth referred to as JW18TET, was 0.27% and 0.68% respectively, which are not significantly different according to Chi square tests (n = 1876 and 2339). Furthermore, no significant difference was observed in the frequency of binucleate cells between JW18 (9.1%, n = 873) and JW18TET cells (11%, n = 1081). Thus, *Wolbachia* do not exert an obvious influence on the regulatory or structural mechanisms governing progression of the cell cycle in the JW18 cell line. However this analysis does not preclude more subtle cycle cell effects.

**Figure 1:**
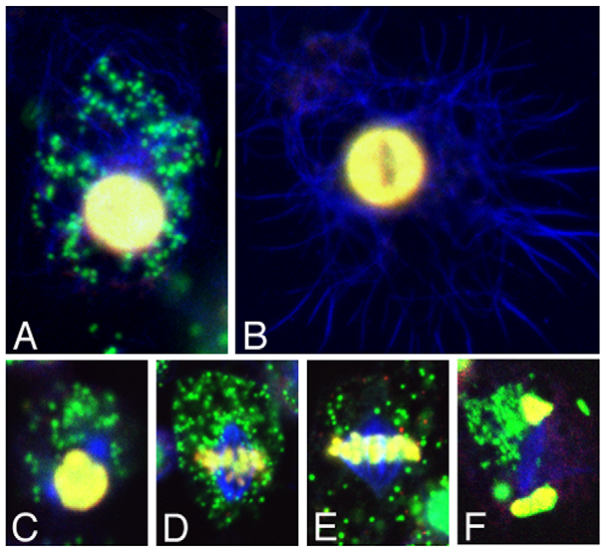
*Wolbachia* distribution in JW18 cells. Interphase of A) JW18 and B) JW18TET cells. Mitotic cells in C) prophase, D) prometaphase, E) metaphase, and F) late anaphase. Blue:Jupiter-GFP. Green: DAPI-labeled *Wolbachia*. Yellow: Histone H1 (A–B) and phospho-histone (C–F). Scale bars: A–B, C–F) 5 µm.

To test whether *Wolbachia* exhibit normal interactions with the host cytoskeleton, we took advantage of the fact that this cell line constitutively expresses a GFP-Jupiter fusion protein that binds to and labels microtubules [52] (Figure 1). During interphase, *Wolbachia* are closely associated with Jupiter-GFP-labeled microtubules (Figure 1A–B, Video S1). Live imaging demonstrates that *Wolbachia* move processively along those interphase microtubules (Video S2), consistent with earlier reports of *Wolbachia*-microtubule interactions [53], [54], [55], [56]. During mitosis, *Wolbachia* were asymmetrically distributed throughout the cytoplasm in 82% of cells (n = 56, Figure 1C–F, Table S1), reminiscent of *Wolbachia* localization patterns observed in embryonic and larval neuroblasts [56]. These data indicate that *Wolbachia* distribution in the JW18 cell line is consistent with that of intact *Drosophila* tissues.

### Automated cell based screening for anti-*Wolbachia* compounds

High-throughput cell-based screens using automated microscopy have proven effective in identifying new compounds targeting specific biological processes [57], [58]. Here we used the JW18 cell line in a 384 well format to screen 2000 compounds from the Spectrum Collection and 2926 compounds from the National Cancer Institute for reduction of *Wolbachia* titer. These libraries include structurally and functionally diverse synthetic compounds, FDA-approved drugs, compounds with biological activity and a set of natural products. After a 5-day incubation period, the cells were fixed, stained, and imaged using automated robotics (Figure S1, Materials and methods). Customized software was used to analyze the images and score the percentage of *Wolbachia*-infected cells in each well. Treatment wells showing significant reduction of *Wolbachia*-infected cells in at least 2 of 3 replicates as compared to the JW18 control and the entire cell population in general were scored as preliminary hits. Compounds known to be generally hazardous were excluded, resulting in a finalized hit list.

**Figure 2:**
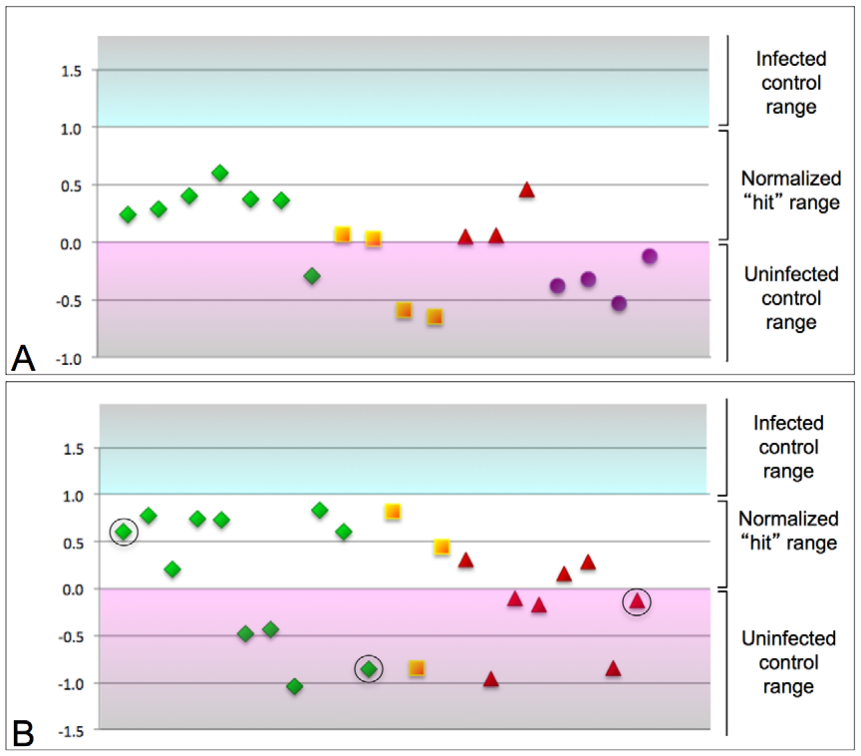
Summary of screening hits. The *Wolbachia* depletion activity of each finalized “hit” compound is displayed as a graphical plot. As the hit range for each plate varied slightly, the range for all plates was normalized to a span from 0–1 to enable a comparative display of all hits. A) Spectrum library hits. B) NCI library hits. Green triangles: hit compounds exhibiting low toxicity. Yellow squares: moderately toxic compounds. Red triangles: toxic compounds. Purple circles: toxicity of the compound unclear. Circled, from left to right: Albendazole-like hits CID5382764, CID5458770, and CID5351210.

18 compounds from the Spectrum library, and 22 compounds from the NCI library exhibited consistent, potent anti-*Wolbachia* activity in the screen (Figure 2, Table S2). A number of these compounds are already known to exert antimicrobial activity, consistent with expectations from the assay. For example, one of the hits CID484401 (totarol acetate) is a known inhibitor of the essential bacterial division protein, FtsZ [59]. CID42640 is a DNA damaging agent that has been shown to inhibit *Mycobacterium tuberculosis* [60]). Significantly, the hit CID313612 is structurally similar to CID42640. CID16524 (pyronin B) is a quaternary ammonium compound, many of which serve as the antimicrobial agents in commercial disinfectants [61]. An additional hit compound, CID6364517 has also been shown in a prior screen to exert antibacterial activity against *Streptococcus pyrogenes* (Pubchem BioAssay AID1900 and AID1915, conducted by the Broad Institute). Furthermore, a number of chemotherapy drugs were identified by the screen: CID30323 (daunorubicin), CID31703 (doxorubicin), CID65348 (epirubicin) and CID4212 (mitoxantrone) were identified as hits, along with the derivitives CID5351490 (cinerubin B) and CID27590 (mitomycin B). Many of these drugs were originally isolated from bacteria in nature. It is presumed that these compounds are used to gain a competitive advantage over neighboring bacteria. As such, this class of drugs is referred to as to “anticancer antibiotics” [62], [63], [64], [65].

**Figure 3:**
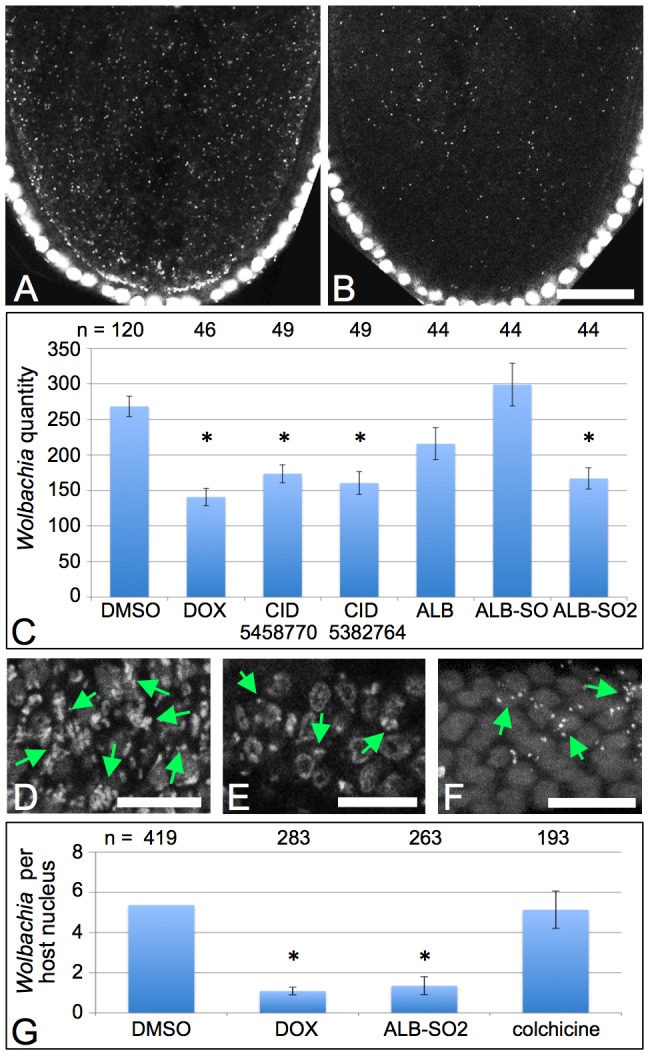
Albendazole-like compounds suppress *Wolbachia* titer in both *D. melanogaster* and *B. malayi*. Propidium iodide staining indicates host DNA as large circles and *Wolbachia* as small puncta. A–C) Assessment of *Wolbachia* titer in stage 10A *D. melanogaster* oocytes. Posterior pole is down. A) DMSO control. B) Doxycycline-treated. C) Average quantity of *Wolbachia* detected in single oocyte focal planes. D–F) *Wolbachia* staining in the *B. malayi* mitotic proliferation zone. Green arrows indicate *Wolbachia* puncta. D) DMSO control. E) Doxycycline-treated. F) Albendazole sulfone-treated. G) Average quantity of *Wolbachia* per host nucleus in the distal ovary of treated *Brugia malayi*. Conditions that significantly deplete *Wolbachia* are indicated by asterisks. Scale bars: A–B) 30 µm. D–F) 10 µm.

Doxycycline (DOX) did not come up as a hit in this screen. This was unexpected as DOX has proven to be an effective anti-*Wolbachia* reagent in lab and clinical settings [6]. However, treating the same *Wolbachia* strain with DOX in the context of *Drosophila* oogenesis revealed marked *Wolbachia* susceptibility to DOX (Figure 3A–C). This suggests that the mechanism accounting for this difference in DOX efficacy is dependent upon properties of the host cell rather than the *Wolbachia* strain. It may be the efflux pumps in the JW18 cell line are particularly effective at expelling DOX. Alternatively, the bacteriostatic effects of DOX may not readily be detected in our assay because of the relative growth rates of *Wolbachia* and the JW18 host cells. Regardless of the mechanism, it is not general, as this screen yielded a number of new potent anti-*Wolbachia* compounds.

**Figure 4:**
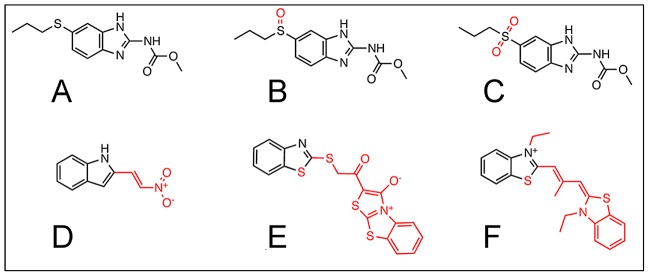
Chemical Structures of Albendazole, Albendazole metabolites and the Albendazole-like NCI hit compounds. Structural differences from the parent compound are indicated in red. A) ALB. B) ALB-SO. C) ALB-SO2. D) CID5382764. E) CID5458770. F) CID5351210.

To further investigate the hit compounds revealed by the screen, we first examined their structures. This revealed that the benzimidazole CID5382764 and the benzthiazoles CID5458770 and CID5351210 share structural similarity to ALB, the widely used, FDA-approved anthelmintic drug known for disrupting helminthic microtubules (Figure 4). The hit compounds were also assessed for cytotoxicity by consulting prior screens run by the NCI against human cancer cell lines and measuring the impact of the compounds on cell proliferation in our own assay. This indicated that 11 of 22 hits from the NCI library and 7 of 18 hits from the Spectrum library exhibit unacceptable toxicity levels, while the remaining 22 hit compounds exhibited low or unknown cytotoxicity (Figure 2, Table S2). By this measure, two of the ALB-like compounds, CID5458770 and CID5382764, were indicated to be non-toxic (Figure 2).

### Albendazole-like compounds reduce *Wolbachia* titer in *Drosophila* oogenesis

*Drosophila* has proven a particularly effective model system for in vivo compound testing of a wide array of biological processes [66]. Here we used the *Drosophila* oocyte as a secondary screen for retesting the ALB-like hits identified in the cell-based screen. This system provides the advantages that the *Wolbachia* strain is the same as in the JW18 cell line, and *Wolbachia* titer in the oocyte is known to greatly increase between stage 3 and 10A, approximately a 40-hour period [54], [67], [68]. Starved adult *Drosophila* were fed yeast paste containing compounds at a concentration of 100 uM for 24 hours. Stage 10A oocytes were fixed and labeled, followed by imaging and quantification of their *Wolbachia* as described in the Materials and Methods. Average titer measurements from single focal planes have been shown to be representative for comparing *Wolbachia* titer between different conditions [67]. DMSO control oocytes exhibited 268+/−14.3 *Wolbachia* within a single oocyte focal plane (Figure 3A–C). Oocytes treated with DOX exhibited 141+/−12.4 *Wolbachia* (p<.001). Treatments with CID5458770 yielded oocytes displaying 174+/−12.7 *Wolbachia* (p<.001). Furthermore, CID5382764-treated oocytes exhibited 161+/−15.9 *Wolbachia* (p<.001, Figure 3). Thus, both of the non-toxic, ALB-like hits identified by the cell screen deplete *Wolbachia* titer in *Drosophila* oogenesis similarly to DOX.

These results motivated us to determine whether ALB and its common metabolites, ALB-SO and ALB-SO2 (Figure 4), also affect *Wolbachia* titer in vivo. *Wolbachia* counts from single oocyte focal planes indicated that ALB and ALB-SO treatments did not significantly affect *Wolbachia* titer, with 216+/−22.5 and 299+/−30.0 *Wolbachia* detected per oocyte, respectively (Figure 3C). However, ALB-SO2-treated oocytes exhibited markedly less *Wolbachia* than the control, with 167+/−14.8 *Wolbachia* evident per oocyte (p<.001, Figure 3). This indicates that the ALB-SO2 metabolite exerts anti-*Wolbachia* effects in *Drosophila*.

### Albendazole sulfone disrupts *Wolbachia* titer in *B. malayi*

To investigate whether the anti-*Wolbachia* effect of ALB-SO2 applies to a disease model, we treated adult *B. malayi* nematodes in vitro. After a 3-day incubation period, *Wolbachia* were imaged in the distal tip region of the *Brugia* ovary referred to as the “mitotic proliferation zone” due to enriched replication of host nuclei in this tissue [69]. *Wolbachia* densely populate this region of the distal ovary in DMSO controls (Figure 3D). By contrast, *Wolbachia* titer is visibly depleted in worms treated with either ALB-SO2 or DOX (Figure 3E–F). Quantitation of *Wolbachia* further supports this observation, with DMSO-treated *Brugia* exhibiting 5.4 *Wolbachia* on average per host nucleus (n = 419 bacteria scored). By contrast, ALB-SO2-treated worms carried significantly fewer bacteria, exhibiting 1.3+/−0.45 *Wolbachia* per host nucleus (n = 263) (p<.001). This depletion was similar to DOX-treated worms, which exhibited 1.1+/−0.19 *Wolbachia* per host nucleus (n = 283) (p<.001). This indicates that the *Wolbachia*-depleting impact of ALB-SO2 extends to *B. malayi*.

### Albendazole sulfone has no impact on microtubule organization in *Drosophila*

A consistent *Wolbachia*-disrupting effect for ALB-SO2 raises the question of the mechanism of action of this metabolite. ALB and other benzimidazoles are known to bind to beta-tubulin and disrupt microtubule polymerization [70], [71], [72], [73]. Previous studies from *Drosophila* have demonstrated that *Wolbachia* titer is affected by host microtubules [54], [67]. This raises the possibility that the reduction of *Wolbachia* titer upon exposure to ALB-SO2 is due to an impact of this compound on microtubule organization. Prior mutant studies identified key amino acids within beta tubulin that are important for the microtubule-disrupting impact of benzimidazoles. Residues N165 and Y200 are thought to form a hydrogen bond that restricts accessibility to a benzimidazole binding site [74]. Interestingly, most of the beta tubulin homologs in *Drosophila* encode the residues that correspond to benzimidazole resistance (Figure S2). This suggests that the *Drosophila* microtubule cytoskeleton should be unaffected by ALB-SO2 treatment.

**Figure 5:**
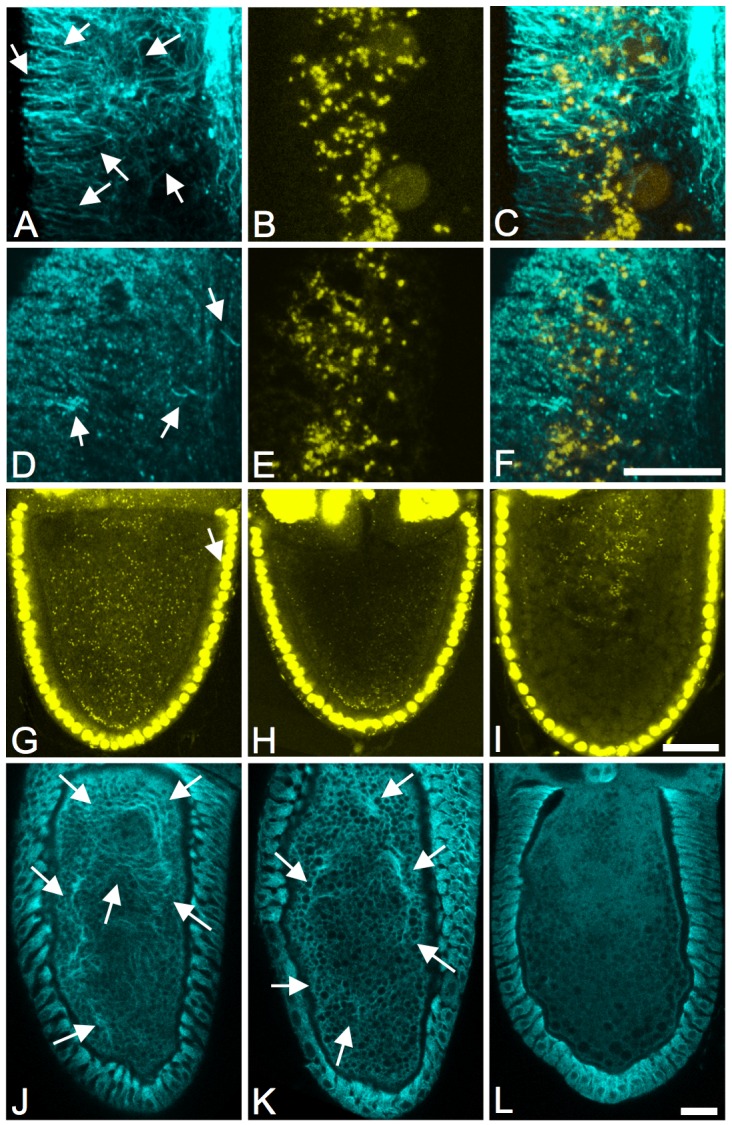
ALB-SO2 visibly disrupts microtubules in *Brugia*, but not in *D. melanogaster*. A–F) Images from the *Brugia* lateral chord. A–C) DMSO control. D–F) ALB-SO2-treated. G–I) Propidium iodide labeling in stage 10A *Drosophila* oocytes. G) DMSO control. H) ALB-SO2-treated. I) Colchicine-treated. J–L) Anti-alpha tubulin staining in stage 10B oocytes. J) DMSO control. K) ALB-SO2-treated. L) Colchicine-treated. Arrows indicate microtubule bundles. Scale bars: A–F) 12 **µ**m. G–L) 30 **µ**m.

To test whether ALB-SO2 affects microtubule organization in *Drosophila* oogenesis, a combination of approaches was used. Intracellular *Wolbachia* localization was examined, taking advantage of the prior finding that *Wolbachia* concentrate at the oocyte posterior cortex in a microtubule-dependent manner [55], [75]. In this study, posterior *Wolbachia* localization was detected in 95% of DMSO controls and 93% of ALB-SO2-treated oocytes (n = 56 and 46, respectively, Figure 5G–H). This differed significantly from oocytes treated with the microtubule-disrupting drug, colchicine, where only 21% exhibited posterior *Wolbachia* localization (p<.001, n = 13, Figure 5I) [55]. The *Drosophila* oocyte cytoskeleton was also directly examined by immunolabeling microtubules [76]. Stage 10B oocytes are known to undergo large-scale, microtubule-dependent cytoplasmic streaming, coincident with formation of microtubule bundles. As cytoplasmic streaming is a highly dynamic process, this bundling varies somewhat between oocytes, and changes within individual oocytes over time [76], [77]. DMSO controls and oocytes treated with ALB-SO2 exhibited microtubule bundling at stage 10B, while the cytoplasm of colchicine-treated stage 10B oocytes was devoid of filamentous structure (Figure 5J–L) [77]. Thus, ALB-SO2 did not affect the overall orientation or structure of the oocyte microtubule cytoskeleton in *Drosophila*, though this compound dramatically decreases *Wolbachia* titer (Figure 3). This indicates that ALB-SO2 reduces *Wolbachia* titer through a microtubule-independent mechanism in *Drosophila*.

### Albendazole sulfone disrupts *Wolbachia* titer in *Brugia* via a microtubule-independent mechanism

The findings above raise the question of whether ALB-SO2 disrupts *Wolbachia* titer independently of microtubules in *B. malayi*, analogous to *Drosophila*. The beta-tubulin genes of *B. malayi* carry the amino acid changes of N165S and Y200F that are associated with susceptibility to benzimidazoles like ALB-SO2 (Figure S2). To assess the impact of ALB-SO2 on *B. malayi* microtubules in vivo, whole-mount immunostaining was performed. Examination of *Brugia* hypodermal chords revealed a dense meshwork of microtubules in DMSO-treated controls (Figure 5A–C). In contrast, treatment with ALB-SO2 disrupted the *Brugia* microtubule cytoskeleton, although linear remnants remained visible throughout the hypodermal chord (Figure 5D–F). This demonstrates that ALB-SO2 disrupts much of the host microtubule cytoskeleton in *B. malayi*.

To next determine whether the titer of *Brugia Wolbachia* relies upon host microtubules, we treated *B. malayi* with colchicine. To verify that colchicine disrupts microtubules in *B. malayi*, immunostaining was performed in the hypodermal chords of colchicine-treated worms as above. This analysis revealed that microtubule structure and organization was largely eliminated by colchicine treatment. To then assess the impact of colchicine on *Wolbachia* titer, *Wolbachia* were imaged in the distal ovary. Interestingly, colchicine-treated worms exhibited an average of 5.1+/−0.93 *Wolbachia* per host nucleus (n = 193 bacteria scored), a value that is not significantly different from its DMSO controls by Chi square test (Figure 3G). This indicates that microtubule disruption has little impact on *Wolbachia* titer in *B. malayi*. This suggests that ALB-SO2 is also unlikely to suppress *Wolbachia* titer through its microtubule-disrupting effects, and alternatively implicates a microtubule-independent mechanism for ALB-SO2 suppression of *Wolbachia* titer in *B. malayi*.

### Albendazole sulfone induces *Wolbachia* elongation in *B. malayi* ovary

**Figure 6:**
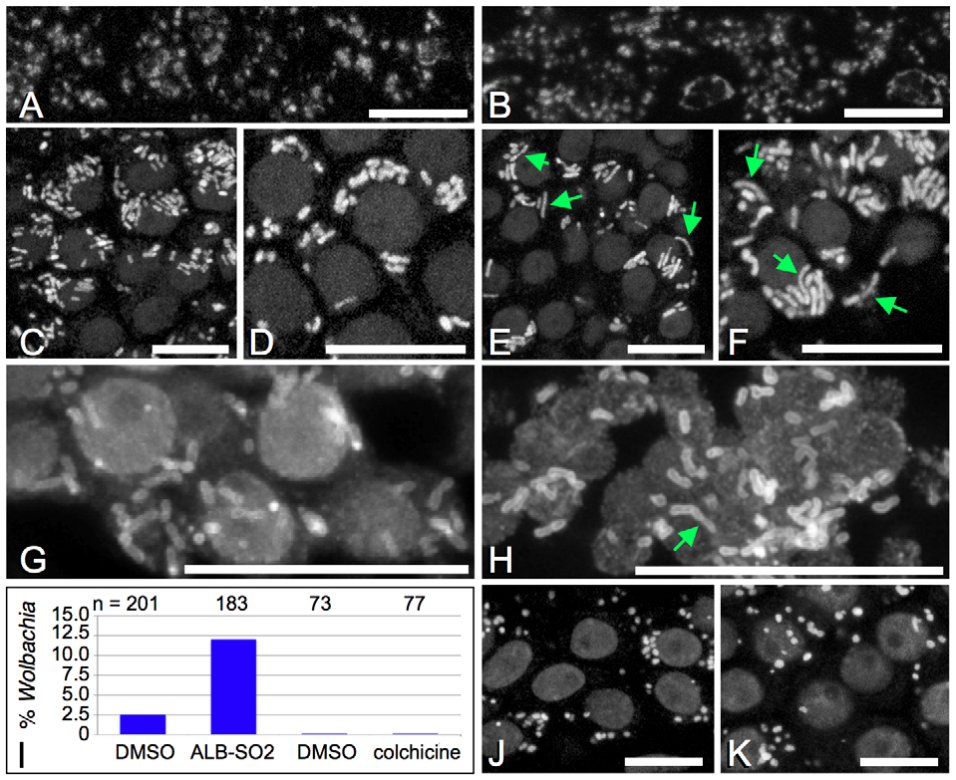
ALB-SO2 treatment induces *Wolbachia* elongation in the *Brugia* ovary, but not in the lateral chords. Green arrows indicate highly elongated *Wolbachia*. A–B) DNA staining is shown in the lateral chords. A) DMSO control. B) ALB-SO2-treated. C–F) DNA staining in the mitotic proliferation zone of the distal ovary. C–D) DMSO control. E–F) ALB-SO2-treated. G–H) FtsZ stain indicates *Wolbachia* (rod shapes) as well as host nuclear background. These panels represent maximum projections of 25 individually captured focal planes. I) Percentage of *Wolbachia* population exceeding 2.5 µm in treated *Brugia* ovaries. J–K) DNA stain in DMSO control (J) vs. a colchicine-treated ovary (K). Scale bars: 10 µm.

To further pursue the mechanism by which ALB-SO2 disrupts *Wolbachia*, we examined its impact on *Wolbachia* morphology in *Brugia* tissue. In the mitotic proliferation zone of the distal ovary, ALB-SO2 treatment corresponded to elongation of *Wolbachia* nucleoids as compared DMSO controls (Figure 6C–F). *Wolbachia* were also immunostained with an antibody raised against *Wolbachia* FtsZ, which crisply defines the boundaries of the *Wolbachia* cytoplasm [78]. The FtsZ staining also revealed an elongated *Wolbachia* morphology in ALB-SO2-treated *Brugia* relative to the DMSO control (Figure 6G–H). Furthermore, quantification of *Wolbachia* length indicated that only 2.5% of *Wolbachia* in the DMSO control exceeded 2.5 µm in length, whereas 17% of *Wolbachia* in ALB-SO2 treated worms exceeded this length (n = 201 and 126, respectively) (p<.001) (Figure 6I). This demonstrates that ALB-SO2 induces *Wolbachia* elongation in the *Brugia* ovary.

*Wolbachia* were also examined in the hypodermal chords, which are syncytial somatic tissues that run along the entire length of the *Brugia* body wall. Comparing *Wolbachia* in DMSO and ALB-SO2-treated hypodermal chords revealed little difference in bacterial morphology (Figure 6A–B). This indicates that *Wolbachia* in the hypodermal chord do not respond to ALB-SO2, in contrast to *Wolbachia* in the distal ovary of *B. malayi*.

### *Wolbachia* elongation occurs independently of host microtubules

A role for ALB-SO2 in inducing *Wolbachia* elongation could be due to an indirect effect from disrupting host microtubules, or through an alternative mechanism. To distinguish between these possibilities, *B. malayi* ovaries were treated with colchicine and assessed for *Wolbachia* length. Examination of DMSO vs. colchicine-treated *Brugia* reveals no obvious differences in *Wolbachia* morphology between conditions (Figure 6J–K). *Wolbachia* length was also quantified, and neither DMSO nor colchicine-treated tissues harbored *Wolbachia* exceeding 2.5 µm in length (Figure 6I). This differs significantly from ALB-SO2-treated *Wolbachia*, which frequently exceeded 2.5 µm in length (p<.001) (Figure 6I). Thus, overall microtubule disruption in *Brugia* did not change *Wolbachia* morphology as was seen for ALB-SO2 treatment. This indicates that ALB-SO2 induces *Wolbachia* elongation independently of host microtubules in *Brugia malayi*.

## Discussion

Here we report the first high-throughput cell-based screen using automated microscopy to identify anti-*Wolbachia* compounds. The cell line used in this screen was established from primary culture preparations from *Wolbachia*-infected *Drosophila* embryos bearing the GFP-tagged microtubule binding protein Jupiter (Figure 1). *Wolbachia* have been stably maintained and transmitted in this line for years. Live analysis of interphase JW18 cells reveals that *Wolbachia* closely associate with and move along microtubules (Video S1), consistent with previous studies showing that *Wolbachia* transport and positioning are microtubule-dependent [54], [55], [56]. Although there is variability from generation to generation, approximately 90% of JW18 cells are infected with variable amounts of bacteria. This is consistent with previous studies of *Wolbachia*-infected *Drosophila*, *Spodoptera frugiperda* and *Aedes albopictus* cell lines showing widely variable bacteria loads per cell [79], [80], [81], [82], [83], [84]. Perhaps this variation in *Wolbachia* load is due in part to asymmetric partitioning of *Wolbachia* in mitotic JW18 cells (Figure 1, Table S1). This same *Wolbachia* strain has previously been reported to segregate asymmetrically in mitotic embryonic *Drosophila* neuroblasts and Aa23 tissue culture cells [56], [82]. In addition, variants have been identified in *D. simulans* in which asymmetric segregation of *Wolbachia* may occur in the germline stem cells of the oocyte [85]. This contrasts with other evidence that *Wolbachia* partition evenly during mitosis in early *Drosophila* embryogenesis and in *A. albopictus* C7-10R tissue culture cells [56], [83]. Future work is needed to address the underlying mechanisms that govern segregation of *Wolbachia* in mitotic cells.

Our automated, image-based screens of 4926 compounds from the NCI and Spectrum libraries revealed 40 compounds that depleted *Wolbachia* titer. Of these, 22 were classified as either non-toxic or of unknown toxicity (Figure 2, Table S2). Among the more characterized hit compounds, we identified totarol acetate, an inhibitor of the key bacterial division protein, FtsZ. This finding is consistent with a previous report describing FtsZ as a potential target for anti-*Wolbachia* therapy [86]. We also identified pararosaniline pamoate and pyrvinium pamoate as *Wolbachia*-depleting hits. These drugs have been employed as antihelmintics to treat patients infected with roundworms like *Enterobius vermicularis* and the trematode *Schistosoma japonicum* [87], [88]. Our result raises an additional possibility that these hosts rely upon endosymbiotic bacteria to support their viability, and an anti-bacterial effect of these drugs thus indirectly compromises the host. No other *Secernentea* outside of the *Filariodea* have been reported to carry *Wolbachia* to date, though the *Rhabditida* order is the only one to have been systematically tested [89]. However, the Trematodes *Acanthatrium oregonense* and *Nanophyetus salmincola* have been reported to harbor the mutualistic bacteria *Neorickettsia risticii* and *N. helminthoseca*, respectively [90]. Furthermore, estradiol was also identified by our screen as a *Wolbachia*-depleting compound. This finding may help to explain why women exhibit symptoms of lymphatic filariasis less often than men in endemic areas, where 10% or fewer of females exhibit symptoms as compared to 10–50% of men [91]. Since *Wolbachia* can induce a TLR-2 and TLR-6-mediated inflammatory response analogous to that observed in lymphatic filariasis patients, the literature invokes a contribution of *Wolbachia* to the pathology of lymphatic filariasis [8], [9], [10], [15], [16], [17]. Perhaps higher estradiol levels in female patients help to suppress *Wolbachia* load, thereby preventing TLR-2/6 induction and development of lymphatic filariasis symptoms.

An examination of the largely uncharacterized hits from the JW18 screen revealed that CID5458770, CID5382764 and CID5351210 structurally resemble ALB (Figure 4). Follow-up studies revealed that the non-toxic ALB-like compounds CID5458770 and CID5382764 exhibited *Wolbachia*-depleting activity in *Drosophila* oogenesis as well. This raised the possibility that ALB or its metabolites may have a similar activity in vivo. These studies surprisingly revealed that the ALB-SO2 metabolite depleted intracellular *Wolbachia* titer in *Drosophila* oogenesis, while ALB and ALB-SO did not (Figure 3). Furthermore, ALB-SO2 disrupts *Wolbachia* titer and morphology and alters host microtubules in *B. malayi* (Figure 3, 5, 6). These findings contrast with routine descriptions in the literature of ALB-SO2 as an inactive metabolite. It appears that ALB-SO2 inactivity has been interpreted from the lower abundance of the ALB-SO2 metabolite in human serum and urine relative to ALB-SO [24], [25], [26] as well as the relatively weaker ability of ALB-SO2 to compete against unlabeled benzimidazoles for binding to *Haemonchus contortus* tubulin in vitro [72]. However, the sequence of *Brugia* beta tubulin predicts susceptibility to benzimidazoles like ALB-SO2 (Figure 4, Figure S2), and evidence from other non-nematode systems supports a role for ALB-SO2 as an active metabolite. An in vitro competitive binding assay using colchicine showed that ALB-SO2 is slightly better at disrupting polymerization of *Ascaris suum* tubulin than either ALB or ALB-SO [92]. Studies from the tapeworm *Echinococcus multilocularis* showed ALB-SO2 to be similarly effective to ALB-SO in inducing structural defects and worm lethality. Other studies of the microsporidian parasites *Encephalitozoon cuniculi*, *E. hellem* and *E. intestinalis* indicated that ALB-SO2 is at least 5 times more effective at inhibiting growth than either ALB or ALB-SO [93]. Building upon those prior findings, this study is the first we are aware of to definitively show an active role for the ALB-SO2 metabolite in filarial nematodes.

A surprising outcome from this study was that ALB-SO2 disrupts *Wolbachia* independently of its effects on host microtubules. Previous studies have shown that host microtubules and microtubule-based motor proteins facilitate replication of *Salmonella* and *Chlamydia* [94], [95], [96], [97], [98], [99], [100], [101]. Prior work in *Drosophila* indicates that normal levels of *Wolbachia* rely in part upon intact host microtubules as well [54], [67]. This study confirms that *Brugia* microtubules are vulnerable to ALB-SO2 in vivo, raising the possibility that ALB-SO2 acts indirectly upon *Wolbachia* through the host cytoskeleton. However, colchicine treatments that eliminate *Brugia* microtubules had no significant impact on *Wolbachia* titer or elongation. Furthermore, ALB-SO2 suppressed *Wolbachia* titer in *Drosophila* even though there was no detectable impact on host microtubules in this system. Thus, ALB-SO2 does not disrupt *Wolbachia* through an influence on the host microtubule cytoskeleton.

The elongation of *Wolbachia* induced by ALB-SO2 suggests a possible mechanism of action for this compound. It has been widely documented that extensive bacterial elongation, referred to as filamentation, ensues when binary fission is disrupted, due to continued growth of the bacteria despite the failure of abscission [73], [102]. Thus, *Wolbachia* elongation in ALB-SO2-treated ovaries suggests that ALB-SO2 is preventing abscission of growing and replicating *Wolbachia*. By contrast, ALB-SO2 treatment had no visible impact on *Wolbachia* length in *Brugia* hypodermal chords. This suggests that the *Wolbachia* bacteria residing within the hypodermal chord are in a non-growing steady state as compared to the proliferating *Wolbachia* population in the distal region of the *B. malayi* ovary. If confirmed, this implies that bacteriostatic drugs may not be effective at clearing *Wolbachia* from the hypodermal chords.

ALB-SO2 could disrupt *Wolbachia* binary fission by targeting a number of different factors. Proper binary fission relies upon assembly of FtsZ filaments at the future fission site, which then leads to recruitment of numerous other factors that help stabilize and constrict the division septum [103]. ALB has been shown to suppress titer and induce filamentation of *Mycobacterium tuberculosis* [104], and numerous studies demonstrate that ALB-like compounds target and disrupt FtsZ in *Staphylococci*, *Escherichia coli* and *M. tuberculosis* [73], [104], [105], [106], [107], [108], [109], [110], [111], [112], [113], [114], [115]. A role for ALB-SO2 in disrupting *Wolbachia* FtsZ function would be consistent with this body of work. However, given staining variability and resolution limits of the *Brugia* system, it is not currently possible to distinguish whether FtsZ concentration or distribution in vivo is significantly different between DMSO and ALB-SO2-treated conditions. An alternative possibility is that ALB-SO2 disrupts binary fission without directly targeting FtsZ. For example, some ALB-like compounds can intercalate into DNA and thus serve as potential DNA damaging agents [73]. DNA damage is known to induce the FtsZ-inhibitor SulA, leading to bacterial filamentation [116], [117]. It is further possible that ALB-SO2 targets other as-yet unrecognized factors that are key to initiation and execution of *Wolbachia* binary fission.

This study has redefined the mechanism by which ALB acts against filarial nematodes. Building upon work by others, the data of this study suggest that ALB administered to humans disrupts *B. malayi* by a two-fold impact. First, ALB and its metabolites disrupt the filarial microtubule cytoskeleton, leading to rapid death of microfilariae. Second, our study indicates that the ALB-SO2 metabolite is directly targeting *Wolbachia*. Certain formulations of ALB were previously shown to lead to *Brugia* sterility in animal models, but the mechanism underlying this effect was unclear [27]. It is possible that microtubule disruption by ALB and its metabolites directly compromises the structure of the adult germline in *Brugia*. However, DOX-based elimination of *Wolbachia* from the female germline has also been shown to induce apoptosis in germline cells [37]. Perhaps ALB-SO2-mediated disruption of *Wolbachia* contributes further to destruction of the *Brugia* germline.

ALB-SO2 may serve as a valuable asset in the campaign against *Wolbachia*-based disease. Used in tandem with conventional antibiotics, ALB-SO2 may shorten the effective treatment time of anti-*Wolbachia* treatments from the current 4 to 6 weeks [5], [6]. It is likely that more potent derivatives of ALB-SO2 may be discovered with enhanced the specificity for *Wolbachia*. Identification of the ALB-SO2 target will enable optimization of the compound to provide a possible alternative route for future treatment of African river blindness and lymphatic filariasis.

## Methods

### Generation of cultured cells

The JW18 cell line bearing the Jupiter-GFP transgene was generated according to the method described in Karpova et al, 2006. 1 to 15 hour old embryos derived from *Wolbachia*-infected flies carrying a Jupiter-GFP transgene were homogenized and plated in flasks. During the next six months of maintenance, five of the initial twenty seed flasks converted into immortal tissue culture lines. The JW18 cell line was selected for further pursuit due to its planar growth pattern and stable abundant *Wolbachia* infection. Cells were maintained at 25–26°C in Sang and Shields media containing 10% fetal bovine serum, split weekly at a 1:3 dilution. A cured version of the JW18 line, referred to as JW18TET, was generated by treating the cells with tetracycline [34], [118], [119]. A Chi-square test was used to test for differences in the frequency of mitosis between JW18 and JW18TET cells.

### Screening approach

Several small chemical libraries were used in this study. The Spectrum Collection (MicroSource Discovery Systems, Inc) of 2000 compounds contains 1000 drugs with known pharmacological properties, 600 natural products, and 400 other bioreactive compounds. The NCI Diversity Set I contains 1990 compounds selected for structural uniqueness and fewer than 5 rotatable bonds. The NCI Mechanistic Set contains 879 compounds, selected for their diversity of impact in human tumor cell lines screened by the NCI. The NCI Challenge Set contains 57 compounds that stood out in the NCI tumor cell screens because of the unusual patterns of cell lethality and resistance induced by these compounds. The stock concentration of the compounds in these libraries was 10 mM. Furthermore, these libraries were reformatted into 384-well plates by the UCSC screening center. Specifically, compounds were placed into columns 3–22 of the plates, leaving columns 1–2 and 23–24 vacant to allow for untreated control wells.

Cells were plated in 384-well, clear bottom plates (Griener Bio-one) pre-coated with 0.5 mg/mL Concanavalin A. JW18 cells were added to 22 columns, and JW18TET cells were added to the remaining 2 columns at a concentration of 6500 cells per well. After the cells adhered to the plates for 4–6 hours, compounds were transferred into 20 columns of JW18 cells in the center of the plate using a Janus MDT pin tool. The final concentration of compound was 100 uM per well. All treatments were distributed into 3 plate replicates.

After a 5-day incubation with the compounds at 25°C, the cells were prepared for imaging. Cells were fixed for 20 minutes in 4% formaldehyde and rinsed with PBS using an automated BioTek liquid handler. All staining solutions were administered using a Multidrop robot, with extensive rinsing between treatments. Mouse anti-histone (MAB052, Millipore) and goat anti-mouse Alexa 594 (Invitrogen) was diluted to 1∶1250 in PBS/0.1% Triton. A stock solution saturated with DAPI was used at a final concentration of 1∶40. After staining, PBS+sodium azide was added to all wells of the plates.

### Screen data analysis

Stained plates were imaged using an ImageXpress Micro system (Molecular Devices, Sunnyvale CA). 10 images were acquired per well at 40× magnification. These images were analyzed using customized analysis software provided by Molecular Devices. The software routine masks any areas where clumps of cells are detected, based upon intensity of the Jupiter-GFP. The boundaries of the remaining cells and their nuclei are recognized based upon the Jupiter-GFP and anti-histone stains. A mask is applied to the nuclei, thus obscuring the histone and DAPI signal from those areas. A threshold for DAPI fluorescence detection is set to detect as much *Wolbachia* as possible in JW18 control cells while minimizing detection of background DAPI signal in JW18TET control cells. The remaining cytoplasmic DAPI, specifically labeling *Wolbachia*, is scored in individual cells to determine whether each cell is infected with *Wolbachia*. The cutoff value distinguishing “infected” from “uninfected” cells is 4000–5000 cytoplasmic DAPI fluorescence units per cell. This is a stringent limit, as values from 10 randomly selected 384-well plates indicate that the untreated JW18TET cells exhibit an average of 2383 +/−78.30 cytoplasmic DAPI fluorescence units per cell as compared to untreated JW18 cells, which exhibit an average of 26146 +/−880.4 DAPI fluorescence units per cell.

A spreadsheet from the data analysis software indicates the quantity of *Wolbachia*-infected cells versus total cells measured in each well. An average and standard deviation were calculated to assess the frequency of *Wolbachia* infection in JW18 and JW18TET control cells, using the descriptive statistics function in SPSS (IBM). These values were used to calculate a Z′ factor for each plate [120]. The Z′ factor represents 1 – (the sum of the standard deviations for each control divided by the absolute value of the difference between mean values for each control.) Z′ factors regarded as acceptable by the field range from 0–1. Our Z′ factors range from 0.2 to 0.65 per plate, verifying that the infected and uninfected controls were clearly distinguishable by the assay. To identify preliminary “hit” compounds that substantially reduce intracellular *Wolbachia* titer, an initial hit range was calculated to lie between the JW18 average infection frequency − 3 standard deviations, and the average JW18TET infection frequency + 3 standard deviations [120]. To enable comparison of hits identified on different treatment plates, the scaling of this initial hit range was next reset to span from 0–1, thus applying a uniform, normalized hit range to all plates.

To further increase the stringency for identifying hits, we also calculated an average infection frequency for all JW18 cells on the plate (treated or not), as most treatment wells are expected to be indistinguishable from untreated controls. Wells that lay within 3 standard deviations of the mean were removed from the hit list. Hit wells identified in only 1 of 3 replicates were also removed from the hit list. From the remaining hit candidates, we further identified compounds designated as hazardous by the National Cancer Institute, which includes alkylating agents, corrosives, carcinogens, explosives, flammables, oxidizers, poisons and known toxins. Any hits falling into these hazard classes were removed from further consideration. The hit compounds not excluded by these stringent criteria were designated as finalized hits.

The cytotoxicity of each hit from the Spectrum and NCI library screens was determined using a stepwise classification process. The Pubchem Bioassay Database was mined to assess the impact of our hits in prior mammalian tumor cell cytotoxicity screens conducted by the NCI. The majority of our hit compounds have already been tested for cytotoxicity in 45–115 screens conducted by the NCI. For those hits, if over 50% of the NCI screens indicated the drug to be cytotoxic, we designated that hit as “toxic” in our listing. If 10%–50% of the NCI screens indicated cytotoxic properties for that compound, we classified it as “moderately toxic.” If less than 10% of NCI screens indicated toxicity, we classified it “non-toxic.” A subset of our hit compounds have been run in 2 or fewer prior NCI screens. For those, hits, a preliminary cytotoxicity designation was assigned based upon the cell density reported by our screen. The average cell density per well was first calculated for each set of treatments, and treatments that failed to shift the cell density of a single well more than +/−33% from the plate average were designated as “non-toxic.” Treatments that increased the average cell density to more that 33% over the average density were classified as “toxicity unclear”, while treatments that reduced average cell density to 33%–66% below the average cell density were classified as “moderately toxic.”

### Drug treatments in *Drosophila* and *Brugia*

ALB (Sigma), ALB-SO and ALB-SO2 (Santa Cruz Biotechnology) were dissolved into DMSO to create 10 mM stock solutions.

The flies used for this study, *w*; *Sp/Cyo*; *Sb/TM6B*, were reared on fly food consisting of 0.5% agar, 7% molasses, 6% cornmeal, and 0.8% killed yeast. Newly eclosed flies were collected, reared for 3 days, starved one day, and then fed compounds of interest for one day. For titer assessment experiments, compounds were diluted to a final concentration of 100 uM in yeast paste. Equivalent amounts of carrier DMSO diluted into these nutrient sources were used as a control.

*Brugia* microfilariae were provided by TRS (Athens, Georgia). These were resuspended in 2 mL tissue culture media containing 50 uM of each compound of interest, except for colchicine, which was administered at 20 uM. Control worms were provided an equivalent dilution of carrier DMSO alone. Microfilariae were incubated with the compounds for 1 day at 37 C with 7.5% CO2. Adult *Brugia* were incubated for 3 days.

### Live imaging of tissue culture cells

Tissue culture chamber slides were coated with 0.5 mg/mL Concanavalin A, followed by addition of JW18 cells. After a 24-hour incubation at 25°C, cells were exposed to Syto-11 (Molecular Probes) for one minute at a dilution of 1∶50,000. Cells were imaged on a Leica SP2 confocal microscope at 100× magnification and 2.75× optical zoom using FITC filters. Images were acquired at 5-second intervals for up to 10 minutes.

### Tissue staining and analysis

A combination of fixation and staining methods was used. Propidium iodide and microtubule staining of *Drosophila* ovarian tissue was also done using established methods [54], [55], [76], [77]. *Brugia* staining was performed as previously described [121]. Briefly, worms were sectioned and fixed in PFA 3.2% for 10 minutes, rinsed in PBS+0.1% Triton-X100, and incubated overnight in RNAseA (10 mg/mL) in PBST, prior a 30 second incubation in a propidium iodide solution (1 mg/mL diluted100X in PBST). Worm fragments were washed for 1 minute in PBST and mounted in DAPI Vectashield mounting medium (Vector Labs). For FtsZ staining, worm fragments were incubated with rabbit anti-FtsZ [78] in PBST after a 1∶500 dilution, after RNAse treatment, washed three times for 10 minutes, before adding a CY5-conjugated anti-rabbit secondary antibody, followed by 3 washes of 10 minutes in PBST and mounting in DAPI Vectashield mounting medium. Microtubules were stained using the monoclonal antibody DM1α (Sigma) at 1∶250 and an Alexa488-conjugated goat anti-mouse secondary antibody (Molecular Probes) was used at 1∶250. Mouse anti-histone H1 (Millipore MAB052) was used at 1∶500, and rabbit anti-phospho-histone H3 (Millipore) was used at 1∶1000.

Data collection was conducted as previously. All tissues were imaged using Leica SP2 and Leica SP5 confocal microscopes. *Wolbachia* were quantified in single focal planes of stage 10A *Drosophila* oocytes using established methods [67]. To measure *Wolbachia* length in the *Brugia* ovaries, 11 images representing a 2 micrometer-thick Z-stack were merged to make a single image, followed by assessment of bacterial length using the Leica SP2 line quantification function. Average *Wolbachia* titer values associated with drug treatments were normalized against their respective DMSO controls as previously to ensure comparability between experiments [67].

Statistical analysis was conducted using established methods. The ANOVA function in SPSS was used to evaluate *Wolbachia* titer differences in *Drosophila* oogenesis and differences in *Wolbachia* length in *B. malayi*. Chi-square tests were used to compare the frequency of *Wolbachia* posterior localization in *Drosophila* oogenesis and to evaluate ratios of *Wolbachia* per host nucleus in the *Brugia* ovary.

## Supporting Information

Figure S1 **Overview of chemical screen strategy.**
(TIF)

Figure S2 **Comparing beta-tubulin residues 165 to 200.** Yellow highlighting showss benzimidazole-resistant residues. Green indicates amino acid changes associated with benzimidazole susceptibility.
(TIF)

Table S1 **Asymmetric distribution of** ***Wolbachia*** **in mitotic JW18 cells.**
(XLS)

Table S2 ***Wolbachia*** **-depleting compounds identified by automated screening assay.**
(XLS)

Video S1 ***Wolbachia*** **colocalize with microtubules in interphase JW18 cells.** Arrows indicate areas of clear overlap between *Wolbachia* and host microtubules. Blue:Jupiter-GFP. Green: DAPI-labeled *Wolbachia*. Yellow: Histone H1.
(AVI)

Video S2 **Asymmetric distribution of** ***Wolbachia*** **in mitotic JW18 cells.** *Wolbachia* labeled with Syto-11 are visible as short, bright green rods. Host microtubules are indicated by Jupiter-GFP as thin, filamentous lines. Movie represents 80 images acquired over 400 seconds of acquisition time. Playback is 40× real time.
(AVI)

## References

[1] Hilgenboecker K, Hammerstein P, Schlattmann P, Telschow A, Werren JH (2008) How many species are infected with Wolbachia? - a statistical analysis of current data. FEMS Microbiol Lett 281: 215–20. 18312577 10.1111/j.1574-6968.2008.01110.x PMC2327208

[2] Serbus LR, Casper-Lindley C, Landmann F, Sullivan W (2008) The Genetics and Cell Biology of *Wolbachia*-Host Interactions. Annu Rev Genet 42: 683–707. 18713031 10.1146/annurev.genet.41.110306.130354

[3] Werren JH, Baldo L, Clark ME (2008) Wolbachia: master manipulators of invertebrate biology. Nat Rev Microbiol 6: 741–751. 18794912 10.1038/nrmicro1969

[4] Harris HL, Braig HR (2003) Sperm chromatin remodelling and Wolbachia-induced cytoplasmic incompatibility in Drosophila. Biochem Cell Biol 81: 229–240. 12897857 10.1139/o03-053

[5] Hoerauf A, Pfarr K, Mand S, Debrah AY, Specht S (2011) Filariasis in Africa–treatment challenges and prospects. Clin Microbiol Infect 17: 977–985. 21722251 10.1111/j.1469-0691.2011.03586.x

[6] Taylor MJ, Hoerauf A, Bockarie M (2010) Lymphatic filariasis and onchocerciasis. Lancet 376: 1175–1185. 20739055 10.1016/S0140-6736(10)60586-7

[7] WHO (2010) World Health Organization Global Programme to Eliminate Lymphatic Filariasis, Progress Report 2000–2009 and Strategic Plan 2010–2020. Geneva: World Health Organization. 78 p.

[8] Debrah AY, Mand S, Marfo-Debrekyei Y, Batsa L, Pfarr K, et al (2009) Reduction in levels of plasma vascular endothelial growth factor-A and improvement in hydrocele patients by targeting endosymbiotic Wolbachia sp. in Wuchereria bancrofti with doxycycline. Am J Trop Med Hyg 80: 956–963. 19478258

[9] Debrah AY, Mand S, Specht S, Marfo-Debrekyei Y, Batsa L, et al (2006) Doxycycline reduces plasma VEGF-C/sVEGFR-3 and improves pathology in lymphatic filariasis. PLoS Pathog 2: e92. 17044733 10.1371/journal.ppat.0020092 PMC1564427

[10] Turner JD, Langley RS, Johnston KL, Gentil K, Ford L, et al (2009) Wolbachia lipoprotein stimulates innate and adaptive immunity through Toll-like receptors 2 and 6 to induce disease manifestations of filariasis. J Biol Chem 284: 22364–22378. 19458089 10.1074/jbc.M901528200 PMC2755959

[11] Brattig NW, Bazzocchi C, Kirschning CJ, Reiling N, Buttner DW, et al (2004) The major surface protein of Wolbachia endosymbionts in filarial nematodes elicits immune responses through TLR2 and TLR4. J Immunol 173: 437–445. 15210803 10.4049/jimmunol.173.1.437

[12] Brattig NW, Buttner DW, Hoerauf A (2001) Neutrophil accumulation around Onchocerca worms and chemotaxis of neutrophils are dependent on Wolbachia endobacteria. Microbes Infect 3: 439–446. 11377205 10.1016/s1286-4579(01)01399-5

[13] Saint Andre A, Blackwell NM, Hall LR, Hoerauf A, Brattig NW, et al (2002) The role of endosymbiotic Wolbachia bacteria in the pathogenesis of river blindness. Science 295: 1892–1895. 11884755 10.1126/science.1068732

[14] Taylor MJ, Cross HF, Bilo K (2000) Inflammatory responses induced by the filarial nematode Brugia malayi are mediated by lipopolysaccharide-like activity from endosymbiotic Wolbachia bacteria. J Exp Med 191: 1429–1436. 10770808 10.1084/jem.191.8.1429 PMC2193140

[15] Hise AG, Daehnel K, Gillette-Ferguson I, Cho E, McGarry HF, et al (2007) Innate immune responses to endosymbiotic Wolbachia bacteria in Brugia malayi and Onchocerca volvulus are dependent on TLR2, TLR6, MyD88, and Mal, but not TLR4, TRIF, or TRAM. J Immunol 178: 1068–1076. 17202370 10.4049/jimmunol.178.2.1068

[16] Turner JD, Langley RS, Johnston KL, Egerton G, Wanji S, et al (2006) Wolbachia endosymbiotic bacteria of Brugia malayi mediate macrophage tolerance to TLR- and CD40-specific stimuli in a MyD88/TLR2-dependent manner. J Immunol 177: 1240–1249. 16818783 10.4049/jimmunol.177.2.1240

[17] Pfarr KM, Debrah AY, Specht S, Hoerauf A (2009) Filariasis and lymphoedema. Parasite Immunol 31: 664–672. 19825106 10.1111/j.1365-3024.2009.01133.x PMC2784903

[18] Korten S, Badusche M, Buttner DW, Hoerauf A, Brattig N, et al (2008) Natural death of adult Onchocerca volvulus and filaricidal effects of doxycycline induce local FOXP3+/CD4+ regulatory T cells and granzyme expression. Microbes Infect 10: 313–324. 18339571 10.1016/j.micinf.2007.12.004

[19] Fernando SD, Rodrigo C, Rajapakse S (2011) Current evidence on the use of antifilarial agents in the management of bancroftian filariasis. J Trop Med 2011: 175941. 21234244 10.1155/2011/175941 PMC3018634

[20] McGarry HF, Plant LD, Taylor MJ (2005) Diethylcarbamazine activity against Brugia malayi microfilariae is dependent on inducible nitric-oxide synthase and the cyclooxygenase pathway. Filaria J 4: 4. 15932636 10.1186/1475-2883-4-4 PMC1173132

[21] Omura S, Crump A (2004) The life and times of ivermectin - a success story. Nat Rev Microbiol 2: 984–989. 15550944 10.1038/nrmicro1048

[22] Moreno Y, Nabhan JF, Solomon J, Mackenzie CD, Geary TG (2010) Ivermectin disrupts the function of the excretory-secretory apparatus in microfilariae of Brugia malayi. Proc Natl Acad Sci U S A 107: 20120–20125. 21041637 10.1073/pnas.1011983107 PMC2993382

[23] Horton J (2009) The development of albendazole for lymphatic filariasis. Ann Trop Med Parasitol 103 Suppl 1: S33–40. 19843396 10.1179/000349809X12502035776595

[24] Marriner SE, Morris DL, Dickson B, Bogan JA (1986) Pharmacokinetics of albendazole in man. Eur J Clin Pharmacol 30: 705–708. 3770064 10.1007/BF00608219

[25] Mirfazaelian A, Dadashzadeh S, Rouini MR (2002) Effect of gender in the disposition of albendazole metabolites in humans. Eur J Clin Pharmacol 58: 403–408. 12242599 10.1007/s00228-002-0488-8

[26] Gottschall DW, Theodorides VJ, Wang R (1990) The metabolism of benzimidazole anthelmintics. Parasitol Today 6: 115–124. 15463313 10.1016/0169-4758(90)90228-v

[27] Gaur RL, Dixit S, Sahoo MK, Khanna M, Singh S, et al (2007) Anti-filarial activity of novel formulations of albendazole against experimental brugian filariasis. Parasitology 134: 537–544. 17078904 10.1017/S0031182006001612

[28] Bockarie MJ, Tavul L, Ibam I, Kastens W, Hazlett F, et al (2007) Efficacy of single-dose diethylcarbamazine compared with diethylcarbamazine combined with albendazole against Wuchereria bancrofti infection in Papua New Guinea. Am J Trop Med Hyg 76: 62–66. 17255231

[29] El Setouhy M, Ramzy RM, Ahmed ES, Kandil AM, Hussain O, et al (2004) A randomized clinical trial comparing single- and multi-dose combination therapy with diethylcarbamazine and albendazole for treatment of bancroftian filariasis. Am J Trop Med Hyg 70: 191–196. 14993632

[30] Hussein O, El Setouhy M, Ahmed ES, Kandil AM, Ramzy RM, et al (2004) Duplex Doppler sonographic assessment of the effects of diethylcarbamazine and albendazole therapy on adult filarial worms and adjacent host tissues in Bancroftian filariasis. Am J Trop Med Hyg 71: 471–477. 15516645

[31] Ismail MM, Jayakody RL, Weil GJ, Nirmalan N, Jayasinghe KS, et al (1998) Efficacy of single dose combinations of albendazole, ivermectin and diethylcarbamazine for the treatment of bancroftian filariasis. Trans R Soc Trop Med Hyg 92: 94–97. 9692166 10.1016/s0035-9203(98)90972-5

[32] Ismail MM, Jayakody RL, Weil GJ, Fernando D, De Silva MS, et al (2001) Long-term efficacy of single-dose combinations of albendazole, ivermectin and diethylcarbamazine for the treatment of bancroftian filariasis. Trans R Soc Trop Med Hyg 95: 332–335. 11491010 10.1016/s0035-9203(01)90257-3

[33] Bosshardt SC, McCall JW, Coleman SU, Jones KL, Petit TA, et al (1993) Prophylactic activity of tetracycline against Brugia pahangi infection in jirds (Meriones unguiculatus). J Parasitol 79: 775–777. 8410553

[34] Bandi C, McCall JW, Genchi C, Corona S, Venco L, et al (1999) Effects of tetracycline on the filarial worms Brugia pahangi and Dirofilaria immitis and their bacterial endosymbionts Wolbachia. Int J Parasitol 29: 357–364. 10221636 10.1016/s0020-7519(98)00200-8

[35] Hoerauf A, Volkmann L, Hamelmann C, Adjei O, Autenrieth IB, et al (2000) Endosymbiotic bacteria in worms as targets for a novel chemotherapy in filariasis. Lancet 355: 1242–1243. 10770311 10.1016/S0140-6736(00)02095-X

[36] Hoerauf A (2000) Targeting of wolbachia endobacteria in litomosoides sigmodontis: comparison of tetracyclines with chloramphenicol, macrolides and ciprofloxacin. Trop Med Int Health 5: 275–279. 10810024

[37] Landmann F, Voronin D, Sullivan W, Taylor MJ (2011) Anti-filarial activity of antibiotic therapy is due to extensive apoptosis after Wolbachia depletion from filarial nematodes. PLoS Pathog 7: e1002351. 22072969 10.1371/journal.ppat.1002351 PMC3207916

[38] Fenn K, Blaxter M (2004) Are filarial nematode Wolbachia obligate mutualist symbionts? Trends Ecol Evol 19: 163–166. 16701248 10.1016/j.tree.2004.01.002

[39] Ghedin E, Wang S, Spiro D, Caler E, Zhao Q, et al (2007) Draft genome of the filarial nematode parasite Brugia malayi. Science 317: 1756–1760. 17885136 10.1126/science.1145406 PMC2613796

[40] Foster J, Ganatra M, Kamal I, Ware J, Makarova K, et al (2005) The Wolbachia genome of Brugia malayi: endosymbiont evolution within a human pathogenic nematode. PLoS Biol 3: e121. 15780005 10.1371/journal.pbio.0030121 PMC1069646

[41] Sironi M, Bandi C, Sacchi L, Di Sacco B, Damiani G, et al (1995) Molecular evidence for a close relative of the arthropod endosymbiont Wolbachia in a filarial worm. Mol Biochem Parasitol 74: 223–227. 8719164 10.1016/0166-6851(95)02494-8

[42] Hoerauf A, Mand S, Volkmann L, Buttner M, Marfo-Debrekyei Y, et al (2003) Doxycycline in the treatment of human onchocerciasis: Kinetics of Wolbachia endobacteria reduction and of inhibition of embryogenesis in female Onchocerca worms. Microbes Infect 5: 261–273. 12706439 10.1016/s1286-4579(03)00026-1

[43] Hoerauf A, Mand S, Adjei O, Fleischer B, Buttner DW (2001) Depletion of wolbachia endobacteria in Onchocerca volvulus by doxycycline and microfilaridermia after ivermectin treatment. Lancet 357: 1415–1416. 11356444 10.1016/S0140-6736(00)04581-5

[44] Hoerauf A, Specht S, Marfo-Debrekyei Y, Buttner M, Debrah AY, et al (2009) Efficacy of 5-week doxycycline treatment on adult Onchocerca volvulus. Parasitol Res 104: 437–447. 18850111 10.1007/s00436-008-1217-8

[45] Hoerauf A, Specht S, Buttner M, Pfarr K, Mand S, et al (2008) Wolbachia endobacteria depletion by doxycycline as antifilarial therapy has macrofilaricidal activity in onchocerciasis: a randomized placebo-controlled study. Med Microbiol Immunol 197: 295–311. 17999080 10.1007/s00430-007-0062-1 PMC2668626

[46] Taylor MJ, Makunde WH, McGarry HF, Turner JD, Mand S, et al (2005) Macrofilaricidal activity after doxycycline treatment of Wuchereria bancrofti: a double-blind, randomised placebo-controlled trial. Lancet 365: 2116–2121. 15964448 10.1016/S0140-6736(05)66591-9

[47] Debrah AY, Mand S, Marfo-Debrekyei Y, Batsa L, Pfarr K, et al (2007) Macrofilaricidal effect of 4 weeks of treatment with doxycycline on Wuchereria bancrofti. Trop Med Int Health 12: 1433–1441. 18076549 10.1111/j.1365-3156.2007.01949.x

[48] Boussinesq M, Gardon J, Gardon-Wendel N, Chippaux JP (2003) Clinical picture, epidemiology and outcome of Loa-associated serious adverse events related to mass ivermectin treatment of onchocerciasis in Cameroon. Filaria J 2 Suppl 1: S4. 14975061 10.1186/1475-2883-2-S1-S4 PMC2147657

[49] Turner JD, Tendongfor N, Esum M, Johnston KL, Langley RS, et al (2010) Macrofilaricidal activity after doxycycline only treatment of Onchocerca volvulus in an area of Loa loa co-endemicity: a randomized controlled trial. PLoS Negl Trop Dis 4: e660. 20405054 10.1371/journal.pntd.0000660 PMC2854122

[50] Turner JD, Mand S, Debrah AY, Muehlfeld J, Pfarr K, et al (2006) A randomized, double-blind clinical trial of a 3-week course of doxycycline plus albendazole and ivermectin for the treatment of Wuchereria bancrofti infection. Clin Infect Dis 42: 1081–1089. 16575724 10.1086/501351

[51] Szollosi A, Debec A (1980) Presence of Rickettsias in Haploid *Drosophila melanogaster* Cell Lines. Extrait Biologie Cellulaire 38: 129–134.

[52] Karpova N, Bobinnec Y, Fouix S, Huitorel P, Debec A (2006) Jupiter, a new Drosophila protein associated with microtubules. Cell Motil Cytoskeleton 63: 301–312. 16518797 10.1002/cm.20124

[53] Kose H, Karr TL (1995) Organization of Wolbachia pipientis in the Drosophila fertilized egg and embryo revealed by an anti-Wolbachia monoclonal antibody. Mech Dev 51: 275–288. 7547474 10.1016/0925-4773(95)00372-x

[54] Ferree PM, Frydman HM, Li JM, Cao J, Wieschaus E, et al (2005) Wolbachia utilizes host microtubules and Dynein for anterior localization in the Drosophila oocyte. PLoS Pathog 1: e14. 16228015 10.1371/journal.ppat.0010014 PMC1253842

[55] Serbus LR, Sullivan W (2007) A Cellular Basis for Wolbachia Recruitment to the Host Germline. PLoS Pathog 3: e190. 18085821 10.1371/journal.ppat.0030190 PMC2134955

[56] Albertson R, Casper-Lindley C, Cao J, Tram U, Sullivan W (2009) Symmetric and asymmetric mitotic segregation patterns influence Wolbachia distribution in host somatic tissue. J Cell Sci 122: 4570–4583. 19934219 10.1242/jcs.054981 PMC2787466

[57] Mayer TU, Kapoor TM, Haggarty SJ, King RW, Schreiber SL, et al (1999) Small molecule inhibitor of mitotic spindle bipolarity identified in a phenotype-based screen. Science 286: 971–974. 10542155 10.1126/science.286.5441.971

[58] Perrimon N, Friedman A, Mathey-Prevot B, Eggert US (2007) Drug-target identification in Drosophila cells: combining high-throughout RNAi and small-molecule screens. Drug Discov Today 12: 28–33. 17198970 10.1016/j.drudis.2006.10.006

[59] Jaiswal R, Beuria TK, Mohan R, Mahajan SK, Panda D (2007) Totarol inhibits bacterial cytokinesis by perturbing the assembly dynamics of FtsZ. Biochemistry 46: 4211–4220. 17348691 10.1021/bi602573e

[60] Kapitzky L, Beltrao P, Berens TJ, Gassner N, Zhou C, et al (2010) Cross-species chemogenomic profiling reveals evolutionarily conserved drug mode of action. Mol Syst Biol 6: 451. 21179023 10.1038/msb.2010.107 PMC3018166

[61] Tischer M, Pradel G, Ohlsen K, Holzgrabe U (2012) Quaternary ammonium salts and their antimicrobial potential: targets or nonspecific interactions? ChemMedChem 7: 22–31. 22113995 10.1002/cmdc.201100404

[62] Muggia FM, Green MD (1991) New anthracycline antitumor antibiotics. Crit Rev Oncol Hematol 11: 43–64. 1831987 10.1016/1040-8428(91)90017-7

[63] Skladanowski A, Konopa J (1994) Interstrand DNA crosslinking induced by anthracyclines in tumour cells. Biochem Pharmacol 47: 2269–2278. 8031321 10.1016/0006-2952(94)90265-8

[64] Pommier Y, Leo E, Zhang H, Marchand C (2010) DNA topoisomerases and their poisoning by anticancer and antibacterial drugs. Chem Biol 17: 421–433. 20534341 10.1016/j.chembiol.2010.04.012 PMC7316379

[65] Tomasz M, Palom Y (1997) The mitomycin bioreductive antitumor agents: cross-linking and alkylation of DNA as the molecular basis of their activity. Pharmacol Ther 76: 73–87. 9535170 10.1016/s0163-7258(97)00088-0

[66] Bell AJ, McBride SM, Dockendorff TC (2009) Flies as the ointment: Drosophila modeling to enhance drug discovery. Fly (Austin) 3: 39–49. 19164936 10.4161/fly.3.1.7774

[67] Serbus L, Ferreccio A, Zhukova M, McMorris C, Kiseleva E, et al (2011) A feedback loop between *Wolbachia* and the *Drosophila gurken* mRNP complex influences *Wolbachia* titer. J Cell Sci 124: 4299–4308. 22193955 10.1242/jcs.092510 PMC3258112

[68] King RC (1970) Ovarian development in Drosophila melanogaster. New York: Academic Press. 227 p.

[69] Kimble J, Crittenden SL (2005) Germline proliferation and its control. In: Community TCeR, editor. WormBook: doi/10.1895/wormbook.1.13.1, http://www.wormbook.org. 10.1895/wormbook.1.13.1 PMC4781503 18050413

[70] Lacey E (1988) The role of the cytoskeletal protein tubulin in the mode of action and mechanism of drug resistance to benzimidazole carbamates. Int J Parasitol 18: 885–936. 3066771 10.1016/0020-7519(88)90175-0

[71] Borgers M, De Nollin S (1975) Ultrastructural changes in Ascaris suum intestine after mebendazole treatment in vivo. J Parasitol 61: 110–122. 1117352

[72] Lubega GW, Prichard RK (1991) Interaction of benzimidazole anthelmintics with Haemonchus contortus tubulin: binding affinity and anthelmintic efficacy. Exp Parasitol 73: 203–213. 1889474 10.1016/0014-4894(91)90023-p

[73] Schaffner-Barbero C, Martin-Fontecha M, Chacon P, Andreu JM (2011) Targeting the assembly of bacterial cell division protein FtsZ with small molecules. ACS Chem Biol 7: 269–277. 22047077 10.1021/cb2003626

[74] Robinson MW, McFerran N, Trudgett A, Hoey L, Fairweather I (2004) A possible model of benzimidazole binding to beta-tubulin disclosed by invoking an inter-domain movement. J Mol Graph Model 23: 275–284. 15530823 10.1016/j.jmgm.2004.08.001

[75] Veneti Z, Clark ME, Karr TL, Savakis C, Bourtzis K (2004) Heads or tails: host-parasite interactions in the Drosophila-Wolbachia system. Appl Environ Microbiol 70: 5366–5372. 15345422 10.1128/AEM.70.9.5366-5372.2004 PMC520876

[76] Serbus LR, Cha BJ, Theurkauf WE, Saxton WM (2005) Dynein and the actin cytoskeleton control kinesin-driven cytoplasmic streaming in Drosophila oocytes. Development 132: 3743–3752. 16077093 10.1242/dev.01956 PMC1534125

[77] Theurkauf WE, Smiley S, Wong ML, Alberts BM (1992) Reorganization of the cytoskeleton during Drosophila oogenesis: implications for axis specification and intercellular transport. Development 115: 923–936. 1451668 10.1242/dev.115.4.923

[78] Landmann F, Bain O, Martin C, Uni S, Taylor M, et al (2012) Both asymmetric mitotic segregation and cell-to-cell invasion are required for stable germline transmission of *Wolbachia* in filarial nematodes. Biology Open In press. 10.1242/bio.2012737 PMC3509449 23213446

[79] Fenollar F, La Scola B, Inokuma H, Dumler JS, Taylor MJ, et al (2003) Culture and phenotypic characterization of a Wolbachia pipientis isolate. J Clin Microbiol 41: 5434–5441. 14662922 10.1128/JCM.41.12.5434-5441.2003 PMC308996

[80] Dobson SL, Marsland EJ, Veneti Z, Bourtzis K, O'Neill SL (2002) Characterization of Wolbachia host cell range via the in vitro establishment of infections. Appl Environ Microbiol 68: 656–660. 11823204 10.1128/AEM.68.2.656-660.2002 PMC126719

[81] Frentiu FD, Robinson J, Young PR, McGraw EA, O'Neill SL (2010) Wolbachia-mediated resistance to dengue virus infection and death at the cellular level. PLoS One 5: e13398. 20976219 10.1371/journal.pone.0013398 PMC2955527

[82] Voronin DA, Bochernikov AM, Baricheva EM, Zakharov IK, Kiseleva EV (2009) [Influence of Drosophila melanogaster genotype on biological effects of endocymbiont Wolbachia (stamm wMelPop)]. Tsitologiia 51: 335–345. 19505052

[83] Venard CM, Crain PR, Dobson SL (2011) SYTO11 staining vs FISH staining: a comparison of two methods to stain Wolbachia pipientis in cell cultures. Lett Appl Microbiol 52: 168–176. 21214605 10.1111/j.1472-765X.2010.02986.x PMC3078573

[84] O'Neill SL, Pettigrew MM, Sinkins SP, Braig HR, Andreadis TG, et al (1997) In vitro cultivation of Wolbachia pipientis in an Aedes albopictus cell line. Insect Mol Biol 6: 33–39. 9013253 10.1046/j.1365-2583.1997.00157.x

[85] Casper-Lindley C, Kimura S, Saxton DS, Essaw Y, Simpson I, et al (2011) Rapid fluorescence-based screening for Wolbachia endosymbionts in Drosophila germ line and somatic tissues. Appl Environ Microbiol 77: 4788–4794. 21622788 10.1128/AEM.00215-11 PMC3147364

[86] Li Z, Garner AL, Gloeckner C, Janda KD, Carlow CK (2011) Targeting the Wolbachia cell division protein FtsZ as a new approach for antifilarial therapy. PLoS Negl Trop Dis 5: e1411. 22140592 10.1371/journal.pntd.0001411 PMC3226453

[87] Katz M (1977) Anthelmintics. Drugs 13: 124–136. 319991 10.2165/00003495-197713020-00002

[88] Cline BL (1982) Current drug regimens for the treatment of intestinal helminth infections. Med Clin North Am 66: 721–742. 7043129 10.1016/s0025-7125(16)31418-3

[89] Bordenstein SR, Fitch DH, Werren JH (2003) Absence of Wolbachia in Nonfilariid Nematodes. J Nematol 35: 266–270. 19262760 PMC2620650

[90] Lin M, Zhang C, Gibson K, Rikihisa Y (2009) Analysis of complete genome sequence of Neorickettsia risticii: causative agent of Potomac horse fever. Nucleic Acids Res 37: 6076–6091. 19661282 10.1093/nar/gkp642 PMC2764437

[91] WHO (2000) Lymphatic Filariasis, Fact Sheet No. 102. World Health Organization.

[92] Barrowman MM, Marriner SE, Bogan JA (1984) The binding and subsequent inhibition of tubulin polymerization in Ascaris suum (in vitro) by benzimidazole anthelmintics. Biochem Pharmacol 33: 3037–3040. 6487354 10.1016/0006-2952(84)90605-1

[93] Ridoux O, Drancourt M (1998) In vitro susceptibilities of the microsporidia Encephalitozoon cuniculi, Encephalitozoon hellem, and Encephalitozoon intestinalis to albendazole and its sulfoxide and sulfone metabolites. Antimicrob Agents Chemother 42: 3301–3303. 9835533 10.1128/aac.42.12.3301 PMC106041

[94] Ramsden AE, Holden DW, Mota LJ (2007) Membrane dynamics and spatial distribution of Salmonella-containing vacuoles. Trends Microbiol 15: 516–524. 17983751 10.1016/j.tim.2007.10.002

[95] Steele-Mortimer O (2008) The Salmonella-containing vacuole: moving with the times. Curr Opin Microbiol 11: 38–45. 18304858 10.1016/j.mib.2008.01.002 PMC2577838

[96] Rajashekar R, Hensel M (2011) Dynamic modification of microtubule-dependent transport by effector proteins of intracellular Salmonella enterica. Eur J Cell Biol 90: 897–902. 21803443 10.1016/j.ejcb.2011.05.008

[97] Hackstadt T, Scidmore-Carlson MA, Shaw EI, Fischer ER (1999) The Chlamydia trachomatis IncA protein is required for homotypic vesicle fusion. Cell Microbiol 1: 119–130. 11207546 10.1046/j.1462-5822.1999.00012.x

[98] Clausen JD, Christiansen G, Holst HU, Birkelund S (1997) Chlamydia trachomatis utilizes the host cell microtubule network during early events of infection. Mol Microbiol 25: 441–449. 9302007 10.1046/j.1365-2958.1997.4591832.x

[99] Hackstadt T, Rockey DD, Heinzen RA, Scidmore MA (1996) Chlamydia trachomatis interrupts an exocytic pathway to acquire endogenously synthesized sphingomyelin in transit from the Golgi apparatus to the plasma membrane. Embo J 15: 964–977. 8605892 PMC449991

[100] Grieshaber SS, Grieshaber NA, Hackstadt T (2003) Chlamydia trachomatis uses host cell dynein to traffic to the microtubule-organizing center in a p50 dynamitin-independent process. J Cell Sci 116: 3793–3802. 12902405 10.1242/jcs.00695

[101] Capmany A, Damiani MT (2010) Chlamydia trachomatis intercepts Golgi-derived sphingolipids through a Rab14-mediated transport required for bacterial development and replication. PLoS One 5: e14084. 21124879 10.1371/journal.pone.0014084 PMC2989924

[102] Erickson HP (1997) FtsZ, a tubulin homologue in prokaryote cell division. Trends Cell Biol 7: 362–367. 17708981 10.1016/S0962-8924(97)01108-2

[103] Margolin W (2005) FtsZ and the division of prokaryotic cells and organelles. Nat Rev Mol Cell Biol 6: 862–871. 16227976 10.1038/nrm1745 PMC4757588

[104] Slayden RA, Knudson DL, Belisle JT (2006) Identification of cell cycle regulators in Mycobacterium tuberculosis by inhibition of septum formation and global transcriptional analysis. Microbiology 152: 1789–1797. 16735741 10.1099/mic.0.28762-0

[105] White EL, Suling WJ, Ross LJ, Seitz LE, Reynolds RC (2002) 2-Alkoxycarbonylaminopyridines: inhibitors of Mycobacterium tuberculosis FtsZ. J Antimicrob Chemother 50: 111–114. 12096015 10.1093/jac/dkf075

[106] Reynolds RC, Srivastava S, Ross LJ, Suling WJ, White EL (2004) A new 2-carbamoyl pteridine that inhibits mycobacterial FtsZ. Bioorg Med Chem Lett 14: 3161–3164. 15149666 10.1016/j.bmcl.2004.04.012

[107] Margalit DN, Romberg L, Mets RB, Hebert AM, Mitchison TJ, et al (2004) Targeting cell division: small-molecule inhibitors of FtsZ GTPase perturb cytokinetic ring assembly and induce bacterial lethality. Proc Natl Acad Sci U S A 101: 11821–11826. 15289600 10.1073/pnas.0404439101 PMC511058

[108] Sarcina M, Mullineaux CW (2000) Effects of tubulin assembly inhibitors on cell division in prokaryotes in vivo. FEMS Microbiol Lett 191: 25–29. 11004395 10.1111/j.1574-6968.2000.tb09314.x

[109] Susanto C (2010) Design and Synthesis of Novel Benzimidazole Library for the Discovery and Development of the Next Generation Antibacterial Agents. Stony Brook: Stony Brook University. 72 p.

[110] Haydon DJ, Stokes NR, Ure R, Galbraith G, Bennett JM, et al (2008) An inhibitor of FtsZ with potent and selective anti-staphylococcal activity. Science 321: 1673–1675. 18801997 10.1126/science.1159961

[111] Nova E, Montecinos F, Brunet JE, Lagos R, Monasterio O (2007) 4′,6-Diamidino-2-phenylindole (DAPI) induces bundling of Escherichia coli FtsZ polymers inhibiting the GTPase activity. Arch Biochem Biophys 465: 315–319. 17678870 10.1016/j.abb.2007.06.032

[112] Adams DW, Wu LJ, Czaplewski LG, Errington J (2011) Multiple effects of benzamide antibiotics on FtsZ function. Mol Microbiol 80: 68–84. 21276094 10.1111/j.1365-2958.2011.07559.x

[113] Andreu JM, Schaffner-Barbero C, Huecas S, Alonso D, Lopez-Rodriguez ML, et al (2010) The antibacterial cell division inhibitor PC190723 is an FtsZ polymer-stabilizing agent that induces filament assembly and condensation. J Biol Chem 285: 14239–14246. 20212044 10.1074/jbc.M109.094722 PMC2863232

[114] Kumar K, Awasthi D, Lee SY, Zanardi I, Ruzsicska B, et al (2010) Novel trisubstituted benzimidazoles, targeting Mtb FtsZ, as a new class of antitubercular agents. J Med Chem 54: 374–381. 21126020 10.1021/jm1012006 PMC3071426

[115] Czaplewski LG, Collins I, Boyd EA, Brown D, East SP, et al (2009) Antibacterial alkoxybenzamide inhibitors of the essential bacterial cell division protein FtsZ. Bioorg Med Chem Lett 19: 524–527. 19064318 10.1016/j.bmcl.2008.11.021

[116] Bi E, Lutkenhaus J (1993) Cell division inhibitors SulA and MinCD prevent formation of the FtsZ ring. J Bacteriol 175: 1118–1125. 8432706 10.1128/jb.175.4.1118-1125.1993 PMC193028

[117] Cordell SC, Robinson EJ, Lowe J (2003) Crystal structure of the SOS cell division inhibitor SulA and in complex with FtsZ. Proc Natl Acad Sci U S A 100: 7889–7894. 12808143 10.1073/pnas.1330742100 PMC164683

[118] Hoerauf A, Nissen-Pahle K, Schmetz C, Henkle-Duhrsen K, Blaxter ML, et al (1999) Tetracycline therapy targets intracellular bacteria in the filarial nematode Litomosoides sigmodontis and results in filarial infertility. J Clin Invest 103: 11–18. 9884329 10.1172/JCI4768 PMC407866

[119] Genchi C, Sacchi L, Bandi C, Venco L (1998) Preliminary results on the effect of tetracycline on the embryogenesis and symbiotic bacteria (Wolbachia) of Dirofilaria immitis. An update and discussion. Parassitologia 40: 247–249. 10376278

[120] Zhang JH, Chung TD, Oldenburg KR (1999) A Simple Statistical Parameter for Use in Evaluation and Validation of High Throughput Screening Assays. J Biomol Screen 4: 67–73. 10838414 10.1177/108705719900400206

[121] Landmann F, Foster JM, Slatko B, Sullivan W (2010) Asymmetric Wolbachia segregation during early Brugia malayi embryogenesis determines its distribution in adult host tissues. PLoS Negl Trop Dis 4: e758. 20689574 10.1371/journal.pntd.0000758 PMC2910707

