# Supplementary figures and images for: A Cell-Based Screen Reveals that the Albendazole Metabolite, Albendazole Sulfone, Targets Wolbachia
Source: PLoS Pathog. 2012 Sep 20;8(9):e1002922. doi: 10.1371/journal.ppat.1002922 (PMC3447747; doi:10.1371/journal.ppat.1002922)

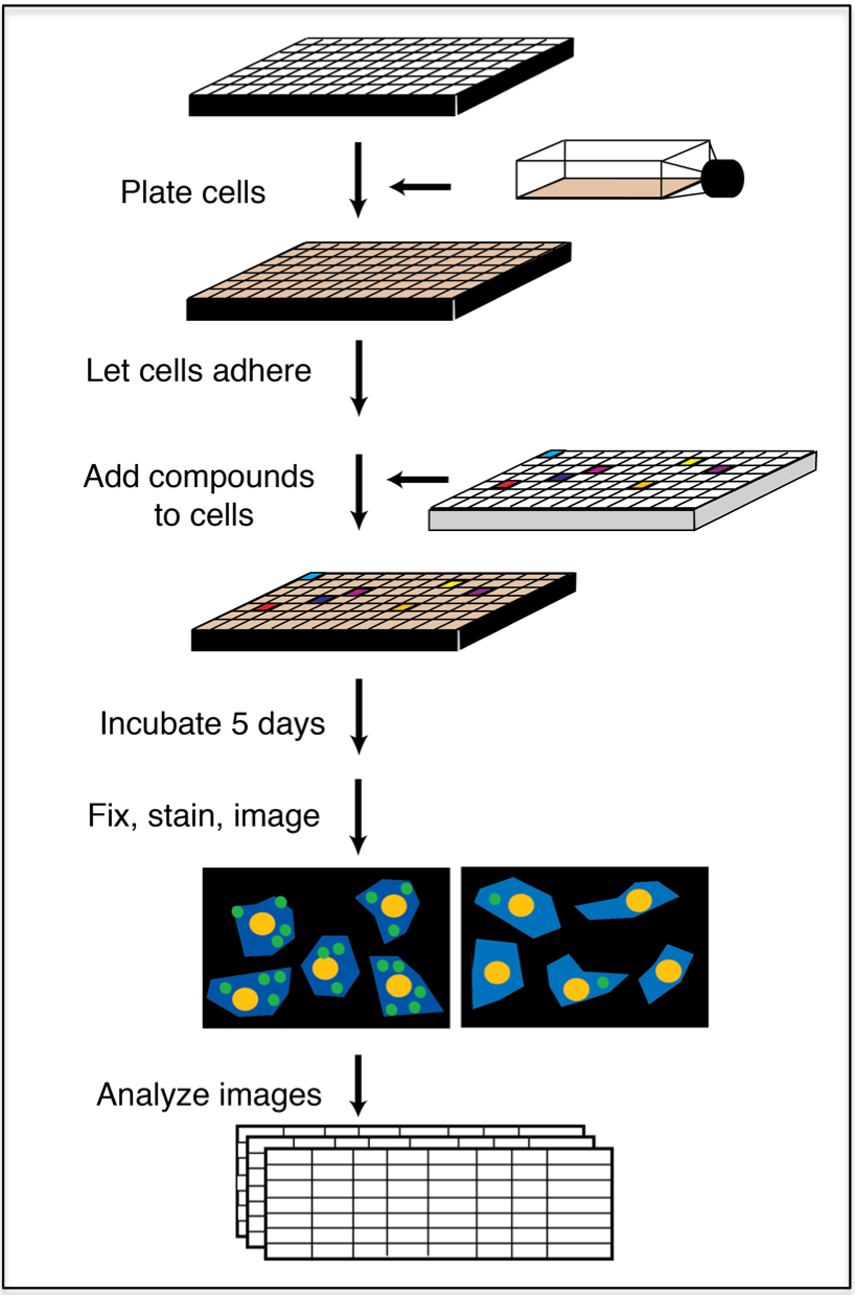

Supplement: Figure S1 — Overview of chemical screen strategy. (TIF) [file ppat.1002922.s001.tif]

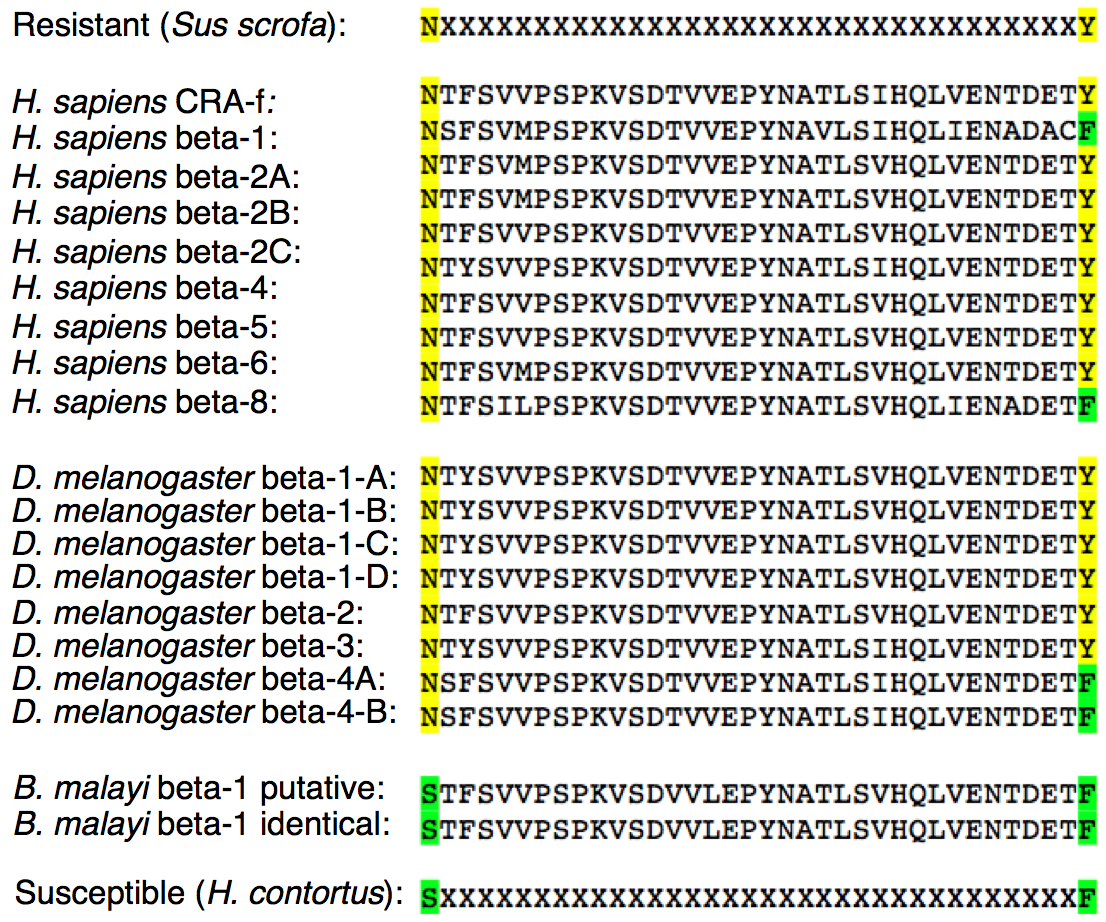

Supplement: Figure S2 — Comparing beta-tubulin residues 165 to 200. Yellow highlighting showss benzimidazole-resistant residues. Green indicates amino acid changes associated with benzimidazole susceptibility. (TIF) [file ppat.1002922.s002.tif]
